# Postoperative ctDNA detection predicts relapse but has limited effects in guiding adjuvant therapy in resectable stage I NSCLC

**DOI:** 10.3389/fonc.2023.1083417

**Published:** 2023-01-20

**Authors:** Bolin Wang, Bing Zou, Shengnan Xu, Chao Zhao, Jinli Pei, Shijie Wang, Kunlong Zhao, Jinming Yu, Jie Liu

**Affiliations:** ^1^ Lung Cancer Center, West China Hospital, Sichuan University, Chengdu, China; ^2^ Department of Radiation Oncology and Shandong Provincial Key Laboratory of Radiation Oncology, Shandong Cancer Hospital and Institute, Shandong First Medical University and Shandong Academy of Medical Sciences, Jinan, Shandong, China; ^3^ Research Unit of Radiation Oncology, Chinese Academy of Medical Sciences, Jinan, Shandong, China

**Keywords:** circulating tumor DNA, NSCLC, prognosis, recurrence, adjuvant therapy

## Abstract

**Background:**

To date, identifying resectable stage I non-small cell lung cancer (NSCLC) patients likely to benefit from adjuvant therapy (ADT) remains a major challenge. Previous studies suggest that circulating tumor DNA (ctDNA) is emerging as a promising biomarker for NSCLC. However, the effectiveness of ctDNA detection in guiding ADT for resectable stage I NSCLC patients remains elusive. This study aimed to elucidate the role of ctDNA detection in estimating prognosis and guiding ADT for resectable stage I NSCLC patients.

**Methods:**

Individual patient data and ctDNA results data were collected from 270 patients across four independent cohorts. The detection of ctDNA was conducted at 3 days to 1 month after surgery. The endpoint for this study was relapse-free survival (RFS) and overall survival (OS).

**Results:**

Of the 270 resectable stage I NSCLC patients, 9 patients with ctDNA-positive and 261 patients with ctDNA-negative. We found that the risk of recurrence was significantly lower in the ctDNA-negative group compared to the ctDNA-positive group(HR=0.11, p<0.0001). However, there is no difference in the risk of death between the two groups (p =0.39). In the ctDNA-positive group, there were no significant differences in RFS between patients who received ADT and patients who did not receive ADT (p =0.58). In the ctDNA-negative group, those who received ADT had a worse RFS in comparison with those who did not receive ADT (HR=2.36, p =0.029). No difference in OS was seen between patients who received ADT and patients who did not receive ADT in both the ctDNA-positive group and the ctDNA-negative group (All p values>0.05). Furthermore, there was no difference in RFS and OS between patients who received chemotherapy-based or tyrosine kinase inhibitor-based ADT and patients who did not receive ADT in both the ctDNA-positive group and the ctDNA-negative group (All p values>0.05).

**Conclusions:**

Postoperative ctDNA detection can be a prognostic marker to predict recurrence but has limited effects in guiding ADT for resectable stage I NSCLC. Future prospective investigations are needed to verify these results.

## Introduction

Non-small cell lung cancer (NSCLC) is the most common type of lung cancer, and a significant effort has been devoted in recent years to improving its diagnosis and treatment (1). To our relief, the rate of patients with lung cancer diagnosed at a localized stage increased from 17% in 2013 to 28% in 2018 according to the Cancer Statistics 2022, representing more patients diagnosed at early stages (1). Stage I NSCLC is a common early stages disease with the most promising potentially cured by lobectomy with mediastinal lymphadenectomy (2, 3). However, there are still 20%–40% of stage I NSCLC patients develop postoperative tumor recurrence (4). Substantial evidence has implicated postoperative minimal residual disease (MRD) is a major contributor to postoperative recurrence, and systemic adjuvant therapy (ADT) after surgery for these patients is a rational strategy to eliminate MRD to improve disease outcomes (5–7). Evidence from Lung Adjuvant Cisplatin Evaluation (LACE) meta-analysis suggests that early-stage NSCLC treated with adjuvant chemotherapy versus no treated has improved only modestly with absolute benefits of 5.4% at 5 years, which suggests “treat many to save few” is commonly encountered in clinical practice (8). For resectable stage I NSCLC patients, the US National Comprehensive Cancer Network (NCCN) guidelines recommend the use of adjuvant chemotherapy with stage IB NSCLC with high-risk factors such as poorly differentiated tumors, vascular invasion, and visceral pleural invasion with acknowledging that “these factors independently may not be an indication” (2). This information highlights the urgent need to identify predictive biomarkers which may assist in selecting patients that may benefit from ADT and thus avoid over- or undertreatment.

Recently, circulating tumor DNA (ctDNA) has emerged as a promising noninvasive biomarker that is highly sought after (9, 10). Multiple prospective studies reported that ctDNA detection has the capacity to identify MRD after therapy in various human tumors (11–14). Evidence from TRACERx studies indicates that ctDNA detection could accurately detect MRD in patients who relapsed before their disease was picked up by standard imaging (15). The findings cited above support further exploring whether ctDNA detection can be employed in treatment stratification and prognosis assessment in NSCLC patients. Several studies show that postoperative detectable ctDNA is associated with a very high risk of recurrence and help to select patients who would most benefit from adjuvant therapy (ADT) in early-stage NSCLC patients (16–18). However, the value of ctDNA in resectable stage I NSCLC is still debated.

Recent evidence shows that postoperative ctDNA positivity is significantly correlated with an increased risk of recurrence and death in stage I NSCLC (18, 19). However, several pieces of evidence suggest that some patients with stage I NSCLC either do not release ctDNA or release ctDNA at frequencies below the limit of detection of current technologies and thus ctDNA detection is unlikely to be widely adopted for NSCLC patients with stage I disease (5). Furthermore, most of the previous work focused on the predictive effect of ctDNA detection in guiding ADT in stage II-III disease due to a few stage I NSCLC patients treated with adjuvant therapy (16, 18). Clinical trials testing postoperative ctDNA status as biomarkers to guide prognostic stratification and ADT decisions for stage I NSCLC patients are underway (NCT05079022; NCT04585477). In view of this, understanding the role of postoperative ctDNA status in stage I NSCLC have important implications for clinical care and future clinical trials.

To address this knowledge gap, we conducted a pooled analysis to investigate the role of ctDNA detection in estimating prognosis and guiding ADT for resectable stage I NSCLC patients. We further analyzed whether postoperative ctDNA detection could be applied to guide different ADT strategies in resectable stage I NSCLC patients to guide clinicians in selecting patients who benefit most from particular ADT regimens.

## Materials and methods

### Patients

Full details of included stage I NSCLC from four cohorts have been published previously (16, 17, 20, 21). The theme of all four included studies was explored in the predictive prognostic value of ctDNA detection in resectable NSCLC patients (16, 17, 20, 21). Data of all patients from four studies were pooled and then ascertained which patients satisfied the necessary inclusion criteria as follows (1): Stage I NSCLC patients, which were defined by pathologic stage or clinical stage, underwent surgical excision of the primary tumor without neoadjuvant therapy; (2) Patients underwent ctDNA detection during collected 3 days to 1 month after surgery with available postoperative ctDNA detection status; (3) Data on ADT and at last follow up and/or date of relapse and death were available. Informed written consent from all study subjects and approval from ethics committees was obtained from each study center.

### Study design

The detection time of ctDNA was during 3 days to 1 month after surgery, given perioperative dynamic changes in ctDNA and the time to initiation of ADT. The ctDNA detection of included cohorts was performed based on next-generation high-throughput sequencing (NGS) and did not consider NGS panel size and content. Detailed methods of ctDNA detection are provided as follows: Peng’s cohort, Circulating Single-molecule Amplification and Resequencing technology consisting of 127 genes; Qiu’s cohort: Automated Triple Groom Sequencing Technology consisting of 139 genes; Xia’s cohort: NGS panel spanning 769 cancer-related genes; Gale’s cohort: Personalized RaDaR™ assays. Postoperative ctDNA status was categorized as detectable (ctDNA-positive) or undetectable (ctDNA-negative) based on the result of a single detection. The endpoint for this study was relapse-free survival (RFS) and overall survival (OS). RFS was defined as the time from surgery to disease recurrence due to any cause. OS was defined as the interval between the time of surgery and death.

### Statistical analysis

Differences in the distribution of categorical variables among different groups were analyzed using Fisher’s exact or chi-squared test. Kaplan–Meier curves of RFS and OS were compared using the standard log-rank test. Univariable and multivariable Cox proportional hazards regression models were used to evaluate proposed prognostic factors. Results are reported as hazard ratios (HR) with 95% confidence intervals (CI). All analyses were performed using SPSS, version 22 (IBM Corp) and R, version 4.1.3 (R Project for Statistical Computing). Two-sided p ≤ 0.05 was considered statistically significant.

## Results

### Overview of patient cohort

**Figure 1:**
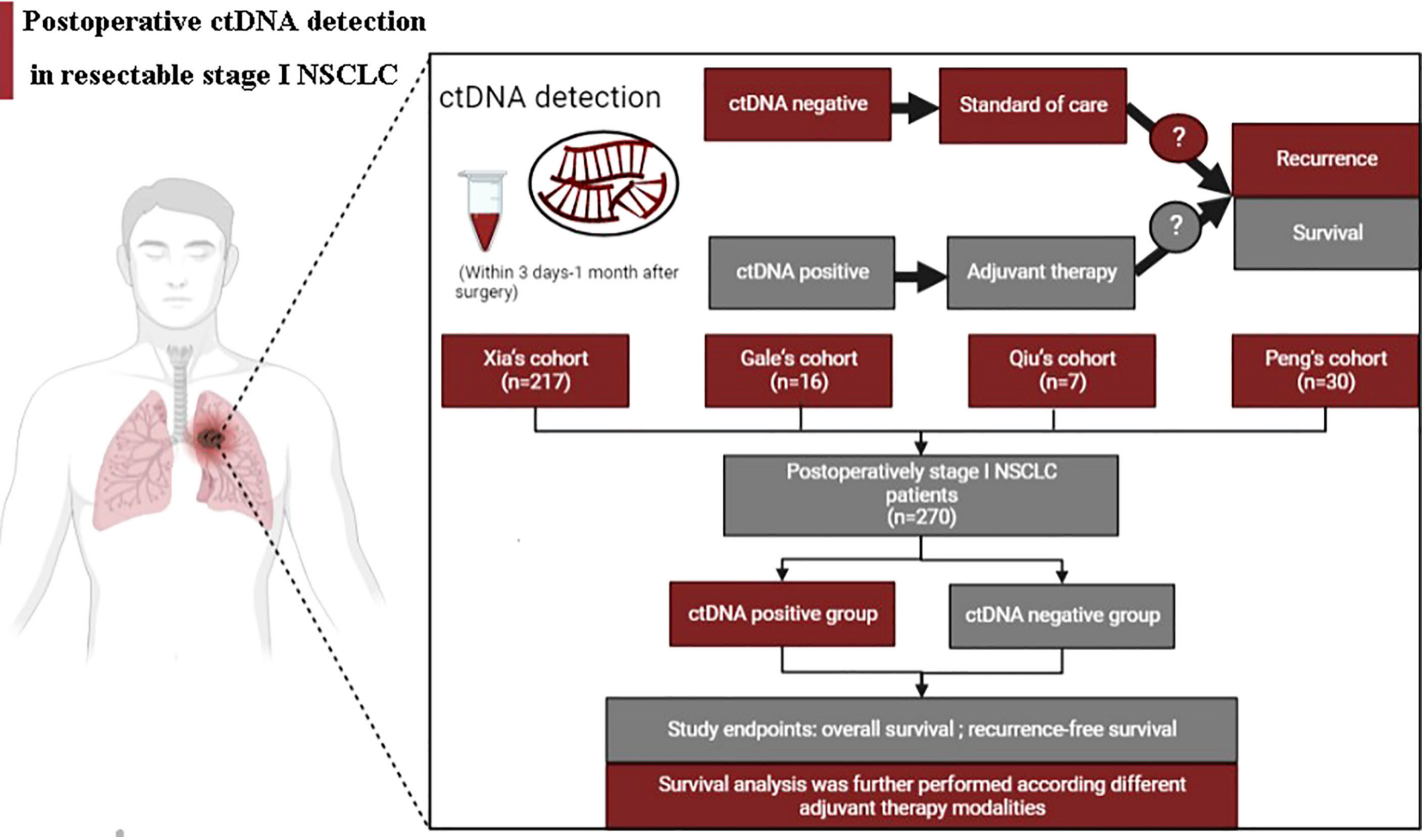
Schematic illustration of working hypotheses and workflow.

**Figure 1** summarizes the working hypotheses and workflow in this study. A total of 270 stage I NSCLC patients from 4 cohorts who had undergone surgical tumor resection and known postoperative ctDNA status were eventually enrolled (16, 17, 20, 21). The large majority of included patients were nonsmokers, pathologically confirmed adenocarcinoma, had T1 stage disease and did not receive postoperative adjuvant therapy. The patients were assigned to the positive and negative groups stratified by postoperative ctDNA status. Of the 270 patients included in this study, 9 had ctDNA-positive (positive rate, 3.33%). The clinicopathological patient characteristics are shown in **Table 1**. There were no significant differences in the baseline clinicopathologic characteristics between ctDNA-positive and ctDNA-negative groups.

**Table 1:** The clinicopathologic characteristics of patients.

Characteristics	ctDNA- (N=261)	ctDNA+ (N=9)	Total (N=270)	p-value
Age (years)				0.32
<60 years	122(46.39%)	6(2.28%)	128(48.67%)	
≥ 60 years	132(50.19%)	3(1.14%)	135(51.33%)	
Sex				0.51
Female	135(51.33%)	6(2.28%)	141(53.61%)	
Male	119(45.25%)	3(1.14%)	122(46.39%)	
Smoker				1
No	180(66.67%)	6(2.22%)	186(68.89%)	
Yes	81(30.00%)	3(1.11%)	84(31.11%)	
Tumor site				1
Left lung	121(47.64%)	4(1.57%)	125(49.21%)	
Right lung	125(49.21%)	4(1.57%)	129(50.79%)	
T stage				0.06
T1	198(77.95%)	4(1.57%)	202(79.53%)	
T2	48(18.90%)	4(1.57%)	52(20.47%)	
Histology subtype				0.42
LUAD	224(82.96%)	7(2.59%)	231(85.56%)	
LUSC	33(12.22%)	2(0.74%)	35(12.96%)	
Other	4(1.48%)	0(0%)	4(1.48%)	
Adjuvant therapy				0.1
No	228(84.44%)	6(2.22%)	234(86.67%)	
Yes	33(12.22%)	3(1.11%)	36(13.33%)	

ctDNA+, ctDNA positive; ctDNA-, ctDNA negative; LUAD, lung adenocarcinoma; LUSC, lung squamous cell carcinoma.

### Postoperative ctDNA detection for predicting recurrence and death

**Figure 2:**
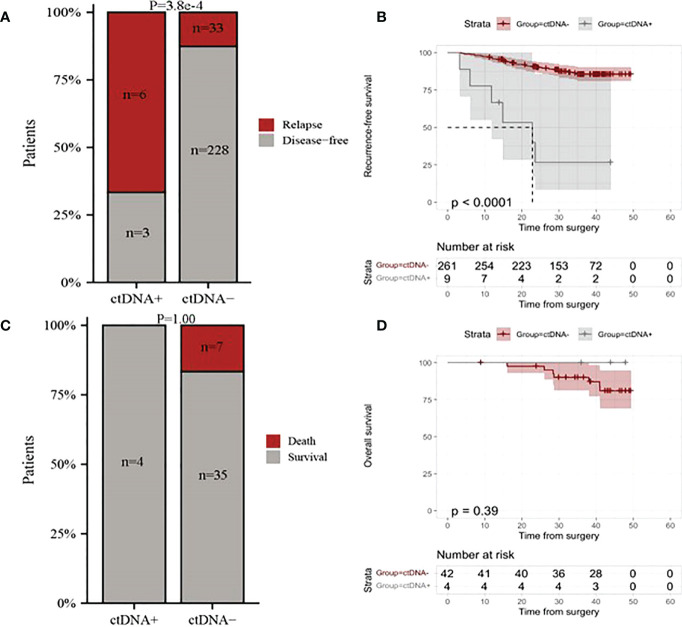
The relationship between postoperative ctDNA status and the risk of recurrence and death in resectable stage I NSCLC patients. **(A)** Comparison of overall relapse proportion between ctDNA-positive and ctDNA-negative patients. **(B)** Kaplan-Meier curves for recurrence-free survival (RFS) for patients with NSCLC stratified by the postoperative ctDNA status. **(C)** Comparison of deaths proportion between ctDNA-positive patients and ctDNA-negative patients. **(D)** Kaplan-Meier curves for overall survival for patients with NSCLC stratified by the postoperative ctDNA status. ctDNA+, ctDNA positive; ctDNA-, ctDNA negative.

**Figure 3:**
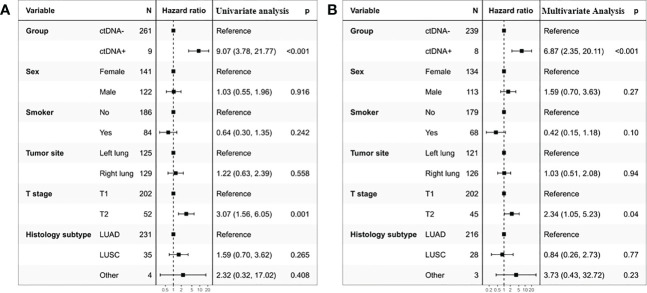
Univariate **(A)** and multivariate **(B)** Cox regression analyses of the association between patient’s characteristics and the probability of recurrence-free survival (RFS) in ctDNA-positive group.

To gain further insight into the potential predictive and prognostic role of postoperative ctDNA detection in resectable stage I NSCLC, we first compared the risk of recurrence between ctDNA-positive and ctDNA-negative groups. Our results indicated that 66.66% (6 of 9) ctDNA-positive patients observed relapse, whereas 12.64% (33 of 261) ctDNA-negative patients experienced relapse (**Figure 2A**, p <0.001). The Kaplan-Meier (KM) curve showed that relapse risks were higher in the ctDNA-positive group compared with the ctDNA-negative group (HR=0.11, 95% CI:0.05-0.26, p<0.0001, **Figure 2B**). To further explore the relationship between ctDNA status and the risk of recurrence, we conducted univariate and multivariate analyses. The results indicated that ctDNA-positive and T stage was an independent prognostic factor with RFS (**Figure 3**). We further investigated the differences in the risk of death between ctDNA-positive and ctDNA-negative groups. We observed that there were no deaths among the 4 ctDNA-positive patients, and 7 out of 42 ctDNA-negative patients died (**Figure 2C**, p =1.00). Furthermore, there were no significant differences in the risk of death between the ctDNA-positive and ctDNA-negative groups (**Figure 2D**, p =0.39).

### Postoperative ctDNA detection for guiding adjuvant therapy

**Figure 4:**
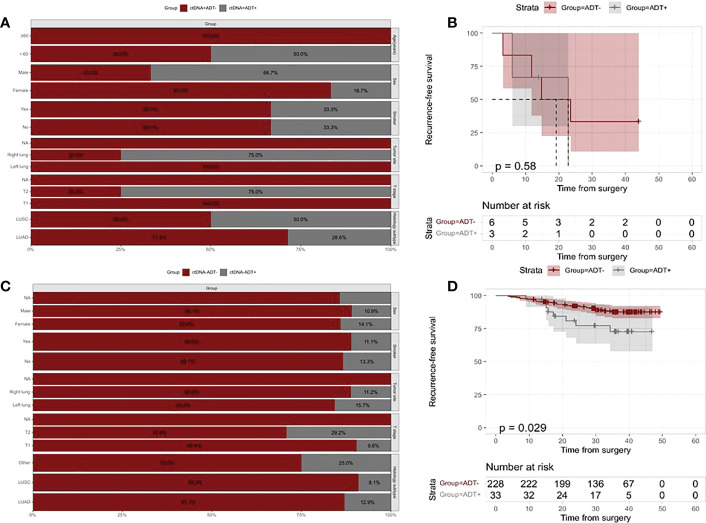
Kaplan–Meier analysis of recurrence-free survival according to ADT and postoperative ctDNA status. **(A)** The distribution of clinicopathologic characteristics for patients who received vs patients who did not receive ADT in the ctDNA-positive group. **(B)** Kaplan-Meier curve showing RFS stratified by ADT in the ctDNA-positive group. **(C)** The distribution of clinicopathologic characteristics for patients who received vs patients who did not receive ADT in the ctDNA-negative group. **(D)** Kaplan-Meier curve showing RFS stratified by ADT in the ctDNA-negative group.

To assess the role of ctDNA detection in directing ADT for resectable stage I NSCLC, we compared prognoses between patients treated with ADT and patients who did not receive ADT in ctDNA-positive and ctDNA-negative groups separately. In the ctDNA-positive group,3 of 9 patients received ADT. Bar chart representing clinical features of patients treated with ADT and patients who did not receive ADT, and the groups were not different in their baseline characteristics (**Figure 4A**). There was no significant difference in relapse risk between patients treated and untreated ADT in the ctDNA-positive group (**Figure 4B**). In the ctDNA-negative group, most of the patients who did not receive ADT had T1 stage disease, and no differences were found in other clinicopathologic characteristics between patients treated and untreated ADT (**Figure 4C**). Our results indicated that ctDNA-negative patients who were treated with ADT were found to be at higher risk of relapse than patients who did not (HR=2.36, 95% CI:1.07-5.36, p=0.029, **Figure 4D**).

**Figure 5:**
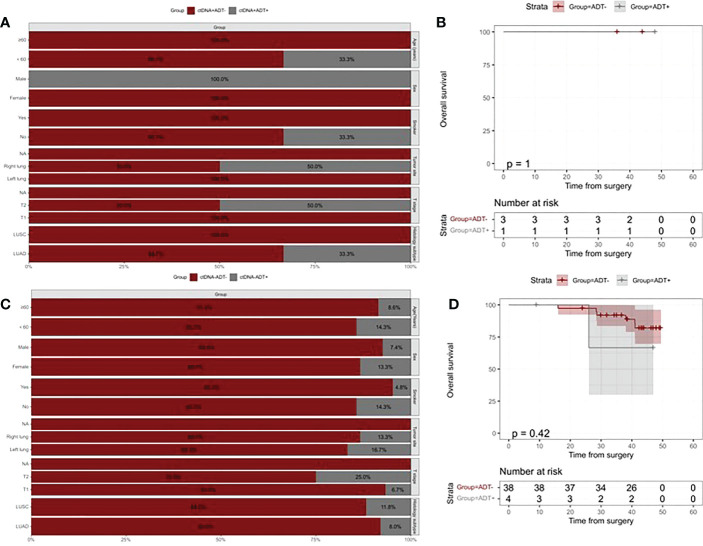
Kaplan–Meier analysis of overall survival according to ADT and postoperative ctDNA status. **(A)** The distribution of clinicopathologic characteristics for patients who received vs patients who did not receive ADT in the ctDNA-positive group. **(B)** Kaplan-Meier curve showing RFS stratified by ADT in the ctDNA-positive group. **(C)** The distribution of clinicopathologic characteristics for patients who received vs patients who did not receive ADT in the ctDNA-negative group. **(D)** Kaplan-Meier curve showing RFS stratified by ADT in the ctDNA-negative group.

We further investigated the risk of mortality according to whether or not received ADT in ctDNA-positive and ctDNA-negative patients. Baseline characteristics of patients who received ADT and those who did not show significant differences in both ctDNA-positive and ctDNA-negative patients **Figures 5A, C**). There were no statistical differences observed in the risk of death between treated and treated ADT in both ctDNA-positive and ctDNA-negative patients (All p values >0.05, **Figures 5B, D**).

### Postoperative ctDNA detection for guiding different adjuvant therapy strategies

**Figure 6:**
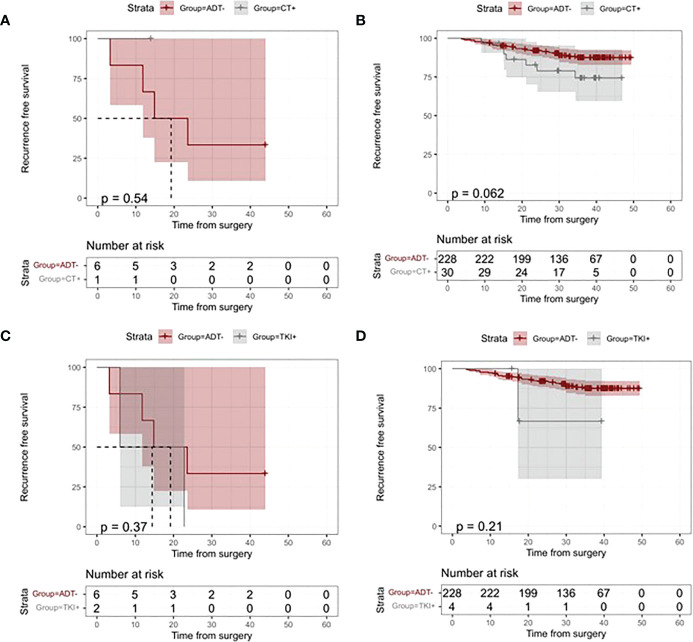
Kaplan–Meier analysis of recurrence-free survival (RFS) according to adjuvant treatment modalities and ctDNA status. **(A)** Kaplan-Meier Curves of RFS for patients who received vs patients who did not receive chemotherapy (CT)-based ADT in the ctDNA-positive group. **(B)** Kaplan-Meier Curves of RFS for patients who received vs patients who did not receive CT-based ADT in the ctDNA negative group. **(C)** Kaplan-Meier Curves of RFS for patients who received vs patients who did not receive adjuvant tyrosine kinase inhibitor (TKI) in the ctDNA-positive group. **(D)**Kaplan-Meier Curves of RFS for patients who received vs patients who did not receive TKI-based ADT in the ctDNA-positive group.

Analyses based on the different ADT types were performed to further understand the role of ctDNA detection in the guided choice of different ADT types. One ctDNA-negative patient who received both CT and TKI ADT was included in CT and TKI ADT subgroup analysis, respectively. **Figure 6** displays no significant difference in risk of relapse between treated and untreated CT-based/TKI-based ADT in both ctDNA-positive and ctDNA-negative patients (All p values >0.05, **Figure 6**). We further analyzed the differences in death risk based on types of adjuvant therapy in ctDNA-positive and ctDNA-negative patients. In the ctDNA-positive group, only one patient received TKI-based ADT. There was no significant risk reduction in patients treated with TKI-based ADT compared with those not treated with ADT (p =1, **Figure 5**). In the ctDNA-negative group, 4 patients received CT-based ADT. We found no difference in death risk between patients treated with CT-based ADT and those who did not treat with ADT (p =0.42, **Figure 5**).

## Discussion

Studies repeatedly demonstrate that ctDNA may serve as a promising biomarker for recurrence (18, 22–25). Notably, several more recent studies have also demonstrated that ctDNA can provide guidance on which patients to treat or not treat with adjuvant chemotherapy injection in resectable NSCLC (17, 18). However, the precise role of postoperative ctDNA detection in stage I NSCLC patients who underwent resection remains unclear. Here, we report a pooled analysis from four cohort studies, focusing on novel endpoints such as the significance of single ctDNA detection, any associations with risk of recurrence and death, and whether they could be used as biomarkers to guide the different types of ADT.

In view of the time to initiate ADT, patient compliance and the association between postsurgical ctDNA status and the prognosis, we focused on single ctDNA detection at postoperative 3 days-1 month in the present study (26, 27). In this study, we first analyzed the postoperative ctDNA-positive detection rate and found only 3.33% of all 270 included patients had ctDNA detected, which was lower than in other studies. The main reasons for the discrepancy can be attributed to variations in the definition of ctDNA-positive. Previous studies examined ctDNA status at multiple time points, and patients with detected ctDNA at one arbitrary time point were defined as ctDNA-positive. However, our findings are based on single ctDNA detection at fixed periods. Furthermore, the association of ctDNA levels with tumor burden has been observed in several studies (15, 26).Thus, stage I NSCLC with low tumor burden had limited ability to detect ctDNA, which is further confirmed by the results shown in our research.

Next, we investigated the prognostic significance of ctDNA status in resectable stage I NSCLC patients. Postoperative ctDNA status was a strong predictive factor for RFS in both univariate and multivariate analysis, further validating previous findings (18, 19). Postoperative ctDNA-positive patients are associated with greater recurrence risk than ctDNA-negative patients. It is worth noting that we observed postoperative ctDNA status was not significantly associated with the risk of death in resectable stage I NSCLC patients. Owing to the low numbers provided by OS information in this study, future studies are required to determine the association between postoperative ctDNA status and death risk.

For additional insights into whether ctDNA status could be used to guide ADT, we compared stage I NSCLC patients’ outcomes stratified by whether they received ADT in the ctDNA-positive group. Our results show no significant difference in relapse and death risk between patients treated and untreated with ADT in the ctDNA-positive group. We further performed a statistical analysis stratified on ADT modalities to further verify and demonstrate the above finding. In the ctDNA-positive group, patients treated with CT-based/TKI-based ADT did not alter the risk for relapse and death compared with patients who did not receive ADT. The above results suggested that postoperative ctDNA status based on single detection is not likely to be useful for helping select patients that can benefit from CT-based/TKI-based ADT.

In the ctDNA-negative group, we observed that patients treated with ADT have a higher recurrence risk than patients untreated with ADT. In this study, ADT was administered following standard clinical guidelines based on prognostic stratification by TNM stage and high-risk factors recommended by the guidelines in the majority of the patient. Thus, most stage IB patients with high-risk factors such as stage T2 disease received ADT, whereas most patients without high-risk factors are free of ADT. Not surprisingly, patients treated with ADT present a higher recurrence risk instead, which is consistent with recent work by Xia et al (16). Furthermore, patients treated with CT-based/TKI-based ADT did not alter the risk for relapse and death compared with patients who did not receive ADT in the ctDNA-negative group.

Ideally, a sensitive enough biomarker could identify high-risk patients who benefit from ADT and low-risk patients who avoid ADT without decreasing the likelihood of prognosis benefit. In light of these findings, postoperative ctDNA status is not a reliable biomarker to guide ADT in resectable stage I NSCLC patients. Evidence from DYNAMIC and TRACER study’s showed stage I NSCLC patients have a low tumor burden and limited capacity to detect ctDNA using current ctDNA approaches, which may diminish sensitivity to identify high-risk patients (15, 26). This may be the primary cause limited impact of ctDNA detection in guiding ADT. In view of this, postoperative single ctDNA detection does not improved treatment selection and how to improve the sensitivity of ctDNA detection in stage I NSCLC is a topic worth exploring.

Three major findings are described in this report. Firstly, the postoperative ctDNA-positive detection rate was lower in stage I NSCLC using current technologies and efforts to improve levels of ctDNA detection in this setting are warranted. Secondly, postoperative ctDNA positive was an independent predictor of relapse but not death. Thirdly, the instructive effects of postoperative ctDNA status on the selection of ADT were limited. Given the lower postoperative ctDNA-positive rate and limited effects of ctDNA detection in guiding ADT, we suggest that ctDNA detection used to guide ADT should be considered cautiously in resectable stage I NSCLC. Several ongoing clinical trials explore the instructive effects of postoperative ctDNA status in guiding ADT for resectable NSCLC patients (NCT05167604; NCT05286957; NCT04966663). We believe this is a novel study that provides the preliminary data for avoiding unnecessary workup and increased costs and may be integrated into future trial designs.

Several limitations should also be considered. Firstly, this study is limited by small sample size, short follow-up and inconsistent ctDNA detection time points. Secondly, the size of the panel design and choice of NGS platform for ctDNA detection could have affected the significance of the results. Thirdly, our result mainly targeted CT-based/TKI-based ADT. Fourthly, we pooled data from four cohorts, which may have introduced bias due to differences in study designs.

## Conclusion

Our findings suggest that postoperative ctDNA detection can be predictive for relapse but has limited effects in guiding ADT in resectable stage I NSCLC. With these findings, ctDNA detection used to guide ADT should be considered cautiously, and reliable prognostic biomarkers are needed to identify patients at high risk for recurrence to guide ADT in resectable stage I NSCLC.

## Data availability statement

The datasets presented in this study can be found in online repositories. The names of the repository/repositories and accession number(s) can be found in the article/supplementary material.

## Ethics statement

Ethical review and approval was not required for the study on human participants in accordance with the local legislation and institutional requirements. Written informed consent to participate in this study was provided by the participants’ legal guardian/next of kin.

## Author contributions

BW, JL, and JY were involved in the literature search, figures, study design, data collection, data analysis, writing and article review. BZ, SX, CZ, JP, SW, and KZ were involved in the literature search and data collection. All authors contributed to the article and approved the submitted version.
